# Behavior change communication activities improve infant and young child nutrition knowledge and practice of neighboring non-participants in a cluster-randomized trial in rural Bangladesh

**DOI:** 10.1371/journal.pone.0179866

**Published:** 2017-06-21

**Authors:** John Hoddinott, Ishita Ahmed, Akhter Ahmed, Shalini Roy

**Affiliations:** 1 Division of Nutritional Sciences, Cornell University, Ithaca, New York, United States of America; 2 Department of Economics, University of Maryland, College Park, Maryland, United States of America; 3 International Food Policy Research Institute (IFPRI), Dhaka, Bangladesh; 4 International Food Policy Research Institute (IFPRI), Washington DC, United States of America; Institut de recherche pour le developpement, FRANCE

## Abstract

**Objective:**

To examine the impact on infant and young child nutrition knowledge and practice of mothers who were neighbors of mothers participating in a nutrition Behavior Change Communication (BCC) intervention in rural Bangladesh.

**Methods:**

We analyzed data from 300 mothers whose neighbor participated in a nutrition BCC intervention and 600 mothers whose neighbor participated in an intervention that did not include BCC. We constructed measures capturing mothers’ knowledge of infant and young child nutrition (IYCN) and measures of food consumption by children 6-24m. The effect on these outcomes of exposure to a neighbor receiving a nutrition BCC intervention was estimated using ordinary least squares and probit regressions. The study was registered with ClinicalTrials.gov (Study ID: NCT02237144).

**Results:**

Having a neighboring mother participate in a nutrition BCC intervention increased non-participant mothers’ IYCN knowledge by 0.17 SD (translating to 0.3 more correct answers). They were 14.1 percentage points more likely to feed their 6-24m children legumes and nuts; 11.6 percentage points more likely to feed these children vitamin A rich fruits and vegetables; and 10.0 percentage points more likely to feed these children eggs. Children of non-participant mothers who had a neighboring mother participate in a nutrition BCC intervention were 13.8 percentage points more likely to meet World Health Organization (WHO) guidelines for minimum diet diversity, 11.9 percentage points more likely to meet WHO guidelines for minimum acceptable diet, and 10.3 percentage points more likely to meet WHO guidelines for minimum meal frequency for children who continue to be breastfed after age 6m. Children aged 0-6m of non-participant mothers who are neighbors of mothers receiving BCC were 7.1 percentage points less likely to have ever consumed water-based liquids.

**Conclusions:**

Studies of nutrition BCC that do not account for information spillovers to non-participants may underestimate its benefits in terms of IYCN knowledge and practice.

## Introduction

A large body of evidence indicates that poor infant and young child nutrition (IYCN) practices contribute to poor pre-school nutrition outcomes [1, 2, 3, 4]. Behavior change communication activities (BCC) are seen as a component of essential actions that will improve IYCN knowledge and practices and lead to improved nutritional outcomes in pre-school children [5]. An overview of studies of effective nutrition BCC activities [6] noted that there have been five systematic reviews [7–11] documenting the impact of BCC on maternal knowledge of correct Infant and Young Child Feeding (IYCF) practices. These five reviews found considerable evidence that nutrition BCC improved knowledge, some evidence that it changed practice, and limited evidence that it led to improved anthropometric outcomes [3]. In Bangladesh, the geographic focus of this paper, Saha and colleagues [12] found that BCC by frontline health workers improved complementary feeding practices, while another study [13] found that BCC reduced diarrhea in pre-school children.

A separate body of literature examines sources of knowledge regarding IYCN practices. A common thread in these literatures is the importance of family members, neighbors, and other mothers and friends as sources of information, in addition to other sources such as health services and the media [14, 15, 16, 17, 18]. Where neighbors are a source of information on IYCN practices, BCC activities directed at mothers may generate information spillovers, with participants passing on this information to neighboring non-participants. If these information spillovers exist, studies of the impact of nutrition BCC on IYCN practices that focus solely on BCC participants may underestimate benefits, due to omitting effects on non-participants. We are unaware of published studies that address this issue. Drawing on qualitative fieldwork in rural Bangladesh, we hypothesized that information provided as part of a high-quality nutrition BCC intervention was being passed on by participants to non-participants. We designed and implemented a quantitative study to address this hypothesis in rural Bangladesh, where stunting remains widespread and IYCN practices sub-optimal [19].

## Materials and methods

### Program description

Between March 2012 and May 2014, the Transfer Modality Research Initiative (TMRI) implemented two randomized controlled trials: one in rural areas of the northwest region (the “North”) where poverty and food insecurity rates are high but food markets function well; and one in rural areas of the coastal southern region (the “South”) where poverty is slightly lower, the climate is hotter, climactic shocks are more frequent, and food markets exist but are less accessible [20]. In the North, in addition to a control group, there were four treatment arms: beneficiaries receiving a cash transfer (“Cash”); a food ration (“Food”); a combination of cash transfer and a food ration (“Cash&Food”); or a cash transfer plus nutrition behavior change communication (“Cash+BCC”). In the south, in addition to a control group, three of these treatment arms—Cash, Food, and Cash&Food—were implemented in an identical way as in the north. However, the fourth treatment arm in the South was different, consisting of the food ration and the same nutrition BCC implemented in the North (“Food+BCC”).

Beneficiaries were extremely poor households with a child aged 0–24 months as of March 2012. Funding available for this intervention enabled payments to be made for 24 months. The intervention was designed in conjunction with the United Nations’ World Food Programme (WFP). WFP was responsible for the management and implementation of the TMRI, including procurement and delivery of transfers and nutrition BCC training. Eco-Social Development Organization (ESDO), an NGO contracted by WFP, was responsible for the field implementation of the transfers, including distributing monthly food and cash transfers, delivering nutrition BCC training sessions, and performing routine monitoring and reporting activities.

The “Cash” arm consisted of a monthly payment of 1,500 Taka (approximately $19 US) per household. The “Food” arm consisted of a monthly food ration of 30 kilograms (kg) of rice, 2 kg of *mosoor* pulse (a type of lentil), and 2 liters of micronutrient-fortified cooking oil. The quantities were chosen so that the value of the food ration was equal to the value of the cash provided in the cash treatment arm as of March 2012. The “Cash&Food” arm provided half of each monthly, specifically 750 Taka, 15kg of rice, 1 kg o*f mosoor* pulse, and 1 liter of micronutrient-fortified cooking oil. All payments were made to mothers.

The “Cash+BCC” arm in the North and the “Food+BCC” arm in the South consisted of the transfer and a suite of intensive nutrition BCC activities. The core activity consisted of a weekly, one-hour group session of the 10 beneficiaries in each village with a trained community nutrition worker (CNW). These sessions covered a defined series of topics: nutrition, diet diversity, and health; handwashing, hygiene, and health; diet diversity and micronutrients; breastfeeding; complementary foods for children 6–24 months; feeding and treatment of children with diarrhea; maternal nutrition; encouraging homestead food production; women’s status and relationships with influential family members (particularly husbands and mothers-in-law) and the wider community. A variety of methods were used to deliver this information including presentations, question and answer, interactive call and answer, songs and chants, practical demonstrations, and role playing. Some sessions were held exclusively for beneficiaries; for others, husbands, mothers-in-law and other influential individuals from beneficiaries’ homes were encouraged to attend. In addition, CNWs made home visits to beneficiaries, to follow up on topics discussed during the group sessions and to discuss specific concerns that mothers might have. While attendance at these BCC sessions was a condition for receipt of transfers, this was a soft condition. When a mother missed a session, the CNW followed up with a home visit to ascertain why the session had been missed, and there were no cases where a beneficiary was dropped from the study for failing to attend sessions. Throughout the intervention, EDSO, WFP, and the study team conducted spot checks on the nutrition BCC to identify problems with delivery and to implement solutions to these. In addition, CNWs and EDSO staff conducted meetings with influential members (for example, village heads, religious leaders, school teachers, local health and family planning staff) of the villages in which the BCC took place to explain the purposes of the nutrition training and to provide them with the information being conveyed to study participants. CNWs received training prior to the start of the intervention, with refresher training undertaken three and 12 months after the intervention began.

Both quantitative and qualitative data collected throughout the intervention indicated that the TMRI was implemented as designed. The nutrition BCC component was successfully implemented. A survey of CNWs fielded in April 2014 included a 14 question quiz on key nutrition messages they were supposed to provide to beneficiaries regarding exclusive breastfeeding; the introduction of complementary foods; the importance of diet diversification; micronutrients; and water, sanitation, and health. The mean score out of 14 was 13.2 in the North and 13.5 in the South. The median household receiving BCC training attended approximately 48 sessions per year in the North and 49 sessions per year in the South, with each session lasting approximately one hour. 83% of respondents reported that, if they missed a session, the nutrition worker followed up with a home visit.

### Study design and participants

Our quantitative study design was inspired by our qualitative fieldwork undertaken as part of the TMRI evaluation in November 2012 and, on a smaller scale, in February 2014. Open-ended questions were asked during key informant interviews and focus group discussions to understand use of transfers, preference for cash or food, how transfers and training affected livelihoods and behaviors, and whether cash and food transfers affect the social/community relations between TMRI participants and non-participants within the communities. A recurrent theme in these interviews and discussions was the impact of the transfer and BCC interventions on women’s ability to interact with others in their village. One respondent noted, “People respect me now. In the past, when I tried to socialize with them, they were not too friendly. They acted as if they were worried I might ask them for a loan.” (Case study #3, [20]). Others noted that the BCC training boosted their family status within the community with neighbors regularly coming over to hear what the family learned during the training sessions, a feature we also observed during our field visits.

Based on these observations, a survey of TMRI neighbors in both the North and South was conducted in April 2014 during the final month of transfer payments, concurrent with the TMRI endline survey. These neighbors were not participants in either randomized trial. We define a “BCC neighbor” household as a household that is: (a) a neighbor of a TMRI household that had received nutrition BCC in addition to their cash or food payment; and (b) had a child aged 0-24m at the time of the survey. We define a “Non-BCC neighbor” household as a household that is: (a) a neighbor of a TMRI household that had received a cash or food payment but no BCC; and (b) had a child aged 0-24m at the time of the survey. Standing at the entrance to the TMRI dwelling or compound, enumerators took a random walk (based on the spin of a pen), walking until they encountered a household with a child aged 0-24m. If the mother of the child consented to an interview, that household was included in the TMRI neighbors study. Enumerators who were conducting the TMRI endline survey also implemented the TMRI neighbors survey. They received three weeks of training that included how to conduct an interview, line-by-line explanation and interpretation of the questionnaires, the flow and skip patterns, definitions, and explanations of how to handle unusual cases and when to contact the supervisor for assistance.

The study received ethical approval from the Institutional Review Board of the International Food Policy Research Institute, Washington DC. The study was also reviewed in Bangladesh by the Ministry of Food and Disaster Management who issued Letters of Authorization to conduct the surveys. Because of high levels of illiteracy in the localities where this study took place, oral consent to participate in the study was received from participants in the TMRI neighbors’ survey, and this oral consent was witnessed and formally recorded. This consent process was approved by the Institutional Review Board of the International Food Policy Research Institute. There were no refusals to participate. The study was registered with ClinicalTrials.gov (Study ID: NCT02237144).

### Sample design

The TMRI evaluation used a cluster randomized controlled trial design. In the North, five upazilas (sub-districts) were selected using simple random sampling from a list of upazilas where in 2010 the proportion of households living below Bangladesh’s lower poverty line was 25 percent or higher. All villages within these five upazilas were listed. Villages classified as peri-urban or villages with fewer than 125 households were dropped. Using a random number generator, each village was assigned a random number. Villages were then sorted in ascending numerical order with the first 275 retained. As described above, in each region, there are four treatment arms and a control group. The first 50 villages were assigned to treatment group 1, the second 50 to treatment group 2, the third 50 villages to treatment group 3, the fourth 50 villages to treatment group 4 and the fifth 50 villages to the control group. The remaining 25 villages were held as a reserve. A complete village census was carried out in each of the 250 selected villages, collecting information on household demographics, a set of poverty indicators, and whether households participate in safety nets and other targeted interventions. Using these data, we constructed a list of households that: (1) were considered poor (i.e., based on the poverty indicators collected, they were estimated to have consumption below Bangladesh’s lower poverty line); (2) had at least one child aged 0–24 months in March 2012; and (3) were not receiving benefits from other safety net interventions. These households were eligible to participate in the study. Using simple random sampling, 10 eligible households were selected from each village. An identical process was used in the southern region to select upazilas, villages and households. S1 and S2 Figs provide flow diagrams of participant participation in the North and South.

The TMRI neighbors study used the 300 villages allocated to the “Cash” treatment arm (50 villages in the North and 50 villages in the South), the “Food” treatment arm (50 villages in the North and 50 villages in the South), the “Cash+BCC” treatment arm (50 villages in the North) and “Food+BCC” (50 villages in the South). To ascertain how many neighbors should be included, power calculations were undertaken drawing on baseline TMRI data and applying the method described in [21]. Setting significance levels at 0.05, statistical power at 0.80, and accounting for intra-cluster correlation of 0.02, a sample of 885 households with a ratio of two “control households” (i.e. TMRI neighbors in the “Cash” and “Food” treatment arms) for every treatment (i.e. TMRI neighbors in the “Cash+BCC” and “Food+BCC” treatment arms) yielded sufficient statistical power to detect a 10 percentage point difference in a two sided test for dichotomous outcomes. With 300 villages in our TMRI neighbors sample, we used the method described above to select three neighbors in each village yielding a total sample of 900 neighbors.

### Measures

Data were collected on household demographics and housing quality, maternal knowledge of infant and young child feeding (IYCF) practices, a one-day recall module on breastfeeding and foods consumed by the child the previous day, and maternal sources of information on IYCF. GIS data were also collected. Questions administered to participants in the neighbors study were identical to a subset of those found in the instrument administered to TMRI participants at endline.

We constructed a z score capturing mothers’ knowledge of IYCF. This is based on seven questions assessing mothers’ knowledge of optimal breastfeeding practices and an additional seven questions on knowledge of complementary foods, foods important for micronutrient intake, and hygiene. To construct this z score, for each mother, the number of correct answers given is summed, and the difference between this sum and the mean number of correct answers in this sample calculated. This difference is divided by the standard deviation for this score.

For children aged 6-24m, data on consumption of food groups was aggregated into seven groups: Grains, roots, tubers; Legumes, nuts; Dairy products; Flesh foods (meat, poultry, fish); Eggs; Vitamin A rich fruits and vegetables; and Other fruit and vegetables. Each was coded as equaling one if the child consumed foods from these groups during the previous day, zero otherwise. Based on World Health Organization (WHO) guidelines [22], we constructed three additional 0/1 variables: whether the child consumed a Minimum meal frequency (for children who continue to be breastfed as is the case for more than 95 percent of the children in our sample); Minimum diet diversity; and Minimum acceptable diet. For children aged 0-6m, we asked whether they had consumed water the previous day, and separately, whether they had consumed water-based liquids (e.g., sugar water) the previous day. We also asked if the child had ever consumed these liquids. These four variables were coded as equaling one if yes, zero if no.

Mother’s knowledge of IYCF and the family of measures of children’s food and liquid consumption are the primary outcomes of this study.

### Statistical analysis

Statistical analyses were conducted in STATA 14.1 (StatCorp LP). Household, maternal, and child characteristics were compared across all treatment and control groups using pairwise t tests. Variables were considered balanced if P >0.05.

Single difference impact estimates were used to measure the impact of being the neighbor of a TMRI household receiving nutrition BCC. These estimates control for child age and sex, whether the mother has any formal schooling, whether the household is female-headed, whether the household head has any formal schooling, and two characteristics of the household dwelling: whether the walls of the dwelling are made of improved materials; and whether the household has an electricity connection. We present estimates based on the pooled sample of North and South households, including a dummy variable equaling one if the household is located in the South.

We used ordinary least squares regressions for the z score for IYCF knowledge. For dichotomous outcomes, we used a probit estimator. Following [23], estimated coefficients are transformed into marginal effects. Standard errors account for clustering at the village level. For IYCF knowledge, impact is considered statistically significant if P ≤ 0.05. We have 14 measures of children’s food and liquid consumption (7 food groups; three aggregate measures and four measures of consumption of liquids). For this family of measures, to correct for multiple testing, we adjusted the p-values to compute two alternate constructions of q-values that control the false detection rate, using the methods described in [24] and [25]. For children’s food and liquid consumption measures, impacts are considered statistically significant if P ≤ 0.05 and furthermore significant with multiple testing corrections if q ≤ 0.05.

We conducted a number of robustness checks. These included assessing whether the impact estimates are sensitive to the inclusion or exclusion of control variables, disaggregating the sample by location, by maternal and child characteristics, and whether the results were sensitive to the use of alternative estimation methods (logits, and linear probability models).

## Results

### Sample profile

900 households (neighbors of TMRI participants) were invited to participate in the study, 450 in the North and 450 in the South. There were no refusals to participate. Table 1 describes the children, mothers, and households in our sample. The average child age was 10.4m. Most mothers, 77%, had some formal schooling, as did 63% of household heads. Few households, 4%, are female-headed. While the majority of households (67%) have dwellings constructed out of improved materials, electricity connections are relatively uncommon (26%). We assessed whether the sample was balanced across these characteristics. There are no significant differences between BCC neighbor and Non-BCC neighbor households across the full sample.

**Table 1:** Unadjusted mean characteristics of children, mothers and households by neighbor status.

	Neighbor of Cash/food + BCC participant (BCC neighbor)	Neighbor of Cash/food only participant (Non-BCC neighbor)
Children, n	300	600
Child age, months	10.4 ±6.3	10.4 ±6.2
Male, %	46.7 ±50.0	50.0 ±50.0
Mother with any schooling, %	77.0 ±42.2	76.0 ±42.7
Household is female headed, %	4.0 ±19.6	5.5 ±22.8
Head with any schooling, %	63.0 ±48.4	57.7 ±49.4
Dwelling walls improved materials, %	67.7 ±46.9	69.7 ±46.0
Has electricity connection, %	26.0 ±43.9	29.8 ±45.8

Notes: Values are means, percentages ±SDs or n. There are no significant differences between BCC neighbor and Non-BCC neighbor households across the full sample. P values available on request.

Table 2 provides reference values for our outcomes on IYCN knowledge and practices among non-BCC neighbor households. On average, mothers in our Non-BCC neighbor households correctly answered 4.1 out of 7 questions regarding optimal breastfeeding practices. They correctly answered 8.6 out of 14 questions regarding optimal breastfeeding, complementary feeding, micronutrients, and WASH.

**Table 2:** Unadjusted mean values of outcome variables for Non-BCC neighbor households.

	IYCF knowledge for mothers in Non-BCC neighbor households
Score (out of 14), IYCF knowledge	8.6 ±1.6
	Day prior to survey, children 6-24m living in Non-BCC neighbor households consumed:
Children, n	426
Grains, roots, tubers, %	88.7 ±31.7
Legumes, nuts, %	29.3 ±45.6
Dairy products, %	20.2 ±40.2
Flesh foods (meat, poultry, fish), %	39.0 ±48.8
Eggs, %	23.9 ±42.7
Vitamin A rich fruits and vegetables, %	47.2 ±50.0
Other fruit and vegetables, %	64.1 ±48.0
Minimum meal frequency (children who are breastfed), %	56.3 ±49.7
Minimum diet diversity, %	40.4 ±49.1
Minimum acceptable diet, %	26.9 ±44.4
	Day prior to survey, children 0-6m living in control households consumed:
Children, n	193
Water, %	32.6 ±47.0
Water based liquids, %	8.8 ±28.4
	Ever, children 0-6m living in control households consumed:
Children, n	174
Water, %	31.0 ±46.4
Water based liquids, %	10.3 ±30.5

Notes: Values are means, percentages ±SDs or n.

Table 2 also provides information on the diets of the children in our Non-BCC neighbor group. Nearly all children (88%) aged 6-24m consumed a starchy grain, root, or tuber. A relatively large fraction consumed Vitamin A rich fruits or vegetables (47%) or some other fruit or vegetable (64%). Between 20 and 40% of children consumed legumes and nuts, dairy products, flesh foods, or eggs. Among children 6-24m whose mothers continued to breastfeed, 56% met WHO guidelines for meal frequency, 40% met WHO guidelines for dietary diversity but only 26% met WHO guidelines for a minimum acceptable diet.

Table 2 additionally reports the consumption of liquids by children 0-6m, on both the day prior to the survey and whether the child had ever consumed these. The consumption of liquids apart from water was relatively infrequent. Across the full sample of control households, 32% of children 0-6m had consumed water the previous day.

### Sources of information

We asked mothers in our neighbor sample about the sources of information on IYCF available to them. They listed a wide variety, including family members, health workers, the media, non-governmental organizations such as BRAC, and neighbors, including those neighbors who are recipients of nutrition BCC through TMRI. 35% of these mothers reported receiving information from neighbors. Only health centers were reported as frequently as a source of information (35%) with the next most frequently reported sources being family (21%) and BRAC (21%). We also asked the mothers in our neighbor sample if they were aware of critical IYCF practices. Awareness of these was high: 93% of mothers reported that they had heard about the importance of starting breastfeeding immediately after delivery or within 1 hour; 92% reported that they had heard about not giving anything except breast milk to your child for six months; 90% reported that they had heard about feeding their baby adequate quantity of family foods in addition to breastmilk from 6–24 months; and 88% reported that they had heard about feeding foods like fish, egg, liver, meat at least once a day for children more than 6 months old. There was less awareness about how to feed a child who has poor appetite (58%) and about how fathers could support mothers to give enough time to the child for proper feeding (42%).

### Effects of being a neighbor of a BCC participant on IYCF knowledge and practice

Being a neighbor of a BCC participant raises the z score on IYCF knowledge by 0.17SD. This effect is statistically significant (P = 0.04). It is equivalent to an additional 0.3 correct answers.

Table 3 reports the effect of being a neighbor of a BCC participant (“BCC neighbor”) on IYCF practices. Relative to neighbors of TMRI households not participating in the nutrition BCC component (“Non-BCC neighbor”), non-participant households who are neighbors of TMRI participants were 14.1 percentage points more likely to feed their 6-24m children legumes and nuts; 11.6 percentage points more likely to feed these children vitamin A rich fruits and vegetables; and 10.0 percentage points more likely to feed these children eggs. These impacts were statistically significant. There were positive, non-significant effects on consumption of dairy products, flesh foods and other fruit and vegetables. Children of non-participant households who are neighbors of TMRI BCC participants were 13.8 percentage points more likely to meet WHO guidelines for minimum diet diversity, 11.9 percentage points more likely to meet WHO guidelines for minimum acceptable diet and 10.3 percentage points more likely to meet WHO guidelines for minimum meal frequency for children who continue to be breastfed after age 6m. Children aged 0-6m of non-participant households who are neighbors of TMRI BCC participants were a statistically significant 7.1 percentage points less likely to have ever consumed water-based liquids. Among these 0-6m children, there were negative, non-significant reductions in consumption of water or water-based liquids in the previous day and in having ever consumed water. Correcting for multiple testing using q-values based on Benjamini, Krieger, and Yekutieli (2006) [24] did not change any of the conclusions about statistical significance; corrections using q-values based on Benjamini and Hochberg (1995) [25] did not change conclusions for six out of seven statistically significant impacts reported and showed borderline significance for the seventh (minimum meal frequency: q = 0.0519).

**Table 3:** Impact of exposure to neighbor’s BCC on infant and young child feeding practices, marginal effects calculated from probit regression with clustered standard errors.

	Marginal effect and Standard Error	P value	Benjamini, Krieger, and Yekutieli (2006) q value	Benjamini and Hochberg (1995) q value
	On day prior to survey, child aged 6–24m consumed			
Legumes, nuts	0.1406 ±0.0419	0.0008	0.0120	0.0110
Vitamin A rich fruits and vegetables	0.1160 ±0.0460	0.0116	0.0340	0.0346
Eggs	0.1001 ±0.0400	0.0123	0.0340	0.0346
Dairy products	0.0504 ±0.0393	0.2001	0.1460	0.2547
Flesh foods (meat, poultry, fish)	0.0384 ±0.0469	0.4129	0.2610	0.4818
Other fruit and vegetables	0.0193 ±0.0417	0.6437	0.2990	0.6437
Grains, roots, tubers	-0.0145 ±0.0247	0.5570	0.2730	0.5999
Minimum diet diversity	0.1379 ±0.0493	0.0051	0.0330	0.0335
Minimum acceptable diet	0.1187 ±0.0442	0.0072	0.0330	0.0335
Minimum meal frequency (children who are breastfed)	0.1034 ±0.0464	0.0259	0.0360	0.0519
Number of observations	634			
	On day prior to survey, child aged 0–6m consumed			
Water	-0.0800 ±0.0614	0.1927	0.1460	0.2547
Water based liquids: teas, sugar water, coffee	-0.0422 ±0.0261	0.1061	0.1030	0.1857
Number of observations	298			
	Ever, child aged 0–6m consumed			
Water	-0.0319 ±0.0615	0.1377	0.1200	0.2142
Water based liquids: teas, sugar water, coffee	-0.0709 ±0.0293	0.0154	0.0350	0.0359
Number of observations	298			

We disaggregated these impact estimates by child sex. We found no outcomes where the differences in the impacts on boys and girls were statistically significant. We disaggregated by maternal characteristics but found no differences based on mother’s schooling or age. Using the GIS data, we assessed whether these effects differed by distance to the neighbor receiving TMRI BCC but found no statistically significant differences. We tested whether the differences between results from the North and South were statistically significant. At the P ≤ 0.05 threshold, we did not reject the null that the impacts in the North and South are equal for any outcome listed in Table 3. For dichotomous outcomes, we re-estimated using a logit estimator and, separately, using a linear probability model; these alternative estimators produced results similar in magnitude to those reported in Table 3.

## Discussion

We find that mothers whose neighbor participated in a nutrition Behavior Change Communication (BCC) intervention scored higher on a measure of IYCN knowledge. The effect size, 0.17SD or 0.3 additional correct answers, is small. This is consistent with our finding that mothers whose neighbor participated in a nutrition Behavior Change Communication (BCC) were more likely to feed their children legumes and nuts, vitamin A rich fruits and vegetables, and eggs. Their 6-24m old children were more likely to meet WHO guidelines for Minimum diet diversity, Minimum acceptable diet, and Minimum meal frequency (for children who continue to be breastfed). Children 0-6m were less likely to consume non-breastmilk liquids. We found positive but insignificant impacts on consumption of dairy and flesh foods.

All these complementary foods are readily available in our study areas. However, they differ significantly in price, with legumes and nuts, vitamin A rich fruits and vegetables, and eggs being relatively inexpensive, and dairy products and flesh foods more expensive. These price differences may explain why we observe differences in impacts across these different food groups. We do not find differential effects based on distances between TMRI participants and their neighbors. However, in these densely populated Bangladeshi villages, neighbors tend to live close by. In a different, more-dispersed, population, these information spillovers might not be as large.

Our study has strengths. We have data on participants in a nutrition BCC intervention and also their neighbors, allowing us to assess the impact of this intervention on non-participants. We obtained data on both IYCN knowledge and practice. Nesting this work in the context of a randomized trial gives us confidence that these effects are causal and not just associational. Our study, however, also has weaknesses. We do not have measures of anthropometric status of children in non-participating households, and so we cannot tell if the magnitude of the impacts on knowledge and practice are sufficient to increase child height or weight. We do not know the precise mechanism underlying these spillover effects. While our qualitative data tells us that mothers in participating households talk with other mothers, the TMRI intervention also included activities such as community meetings. It is possible that these meetings, and not informal mother-to-mother interactions, may have contributed to changed behavior and practice among non-TMRI participants. However, the community meetings were primarily with influential community members such as village heads and religious leaders, rather than with non-TMRI mothers in the community. Moreover, 98% of BCC participant mothers in our endline survey reported passing on the information to other people. Finally, while we have sufficient statistical power to assess impacts across the full sample, we have less power to assess differential impacts across sub-samples.

An implication of these results is that existing studies of the impact of nutrition BCCs may underestimate their benefits. Existing studies focus only on participants in these interventions. Our results show that non-participants may also benefit. A related implication is that the cost-effectiveness of nutrition BCCs may also be understated if it does not take into account these information spillovers. Whether these implications are correct depends, in part, on whether these impacts are found in other nutrition BCC interventions, particularly in localities where populations are more dispersed.

## Supporting information

S1 Fig Flow diagram of participant participation in the North RCT. (DOCX)

S2 Fig Flow diagram of participant participation in the South RCT. (DOCX)

S1 Checklist Consort 2010 checklist. (DOC)

S1 Dataset Data file. (DTA)

S1 Logfile Log file showing results. (LOG)

S1 Appendix Protocol registration. (PDF)
