# Supplementary material for: Behavior change communication activities improve infant and young child nutrition knowledge and practice of neighboring non-participants in a cluster-randomized trial in rural Bangladesh
Source: PLoS One. 2017 Jun 21;12(6):e0179866. doi: 10.1371/journal.pone.0179866 (PMC5479588; doi:10.1371/journal.pone.0179866)
Supplement: S1 Fig — (DOCX) [file pone.0179866.s001.docx]

**Supplementary Figure 1: Flow diagram of participant participation in the North RCT**

2500 Eligible households in 125 villages

500 Households

25 Villages

“Control”

499 Households

25 Villages

“Cash+BCC”

1,500 Taka

BCC

500 Households

25 Villages

“Cash+Food”

750 Taka

15 kg rice

1 kg pulses

1ℓ cooking oil

500 Households

25 Villages

“Food only”

30 kg rice

2 kg pulses

2ℓ cooking oil

499 Households

25 Villages

“Cash only”

1,500 Taka

475 Households

533 Children 6-60m

475 Households

541 Children 6-60m

490 Households

559 Children 6-60m

485 Households

531 Children 6-60m

483 Households

534 Children 6-60m

21 Migrated

1 Refusal

2 Not traced

1 Incomplete data

22 Migrated

0 Refusal

1 Not traced

1 Incomplete data

10 Migrated

0 Refusal

0 Not traced

0 Incomplete data

13 Migrated

2 Refusal

1 Not traced

0 Incomplete data

12 Migrated

3 Refusal

0 Not traced

0 Incomplete data

2498 Households enrolled in study

2 Refusals
