# Supplementary material for: Behavior change communication activities improve infant and young child nutrition knowledge and practice of neighboring non-participants in a cluster-randomized trial in rural Bangladesh
Source: PLoS One. 2017 Jun 21;12(6):e0179866. doi: 10.1371/journal.pone.0179866 (PMC5479588; doi:10.1371/journal.pone.0179866)
Supplement: S2 Fig — (DOCX) [file pone.0179866.s002.docx]

**Supplementary Figure 2: Flow diagram of participant participation in the South RCT**

2500 Eligible households in 125 villages

498 Households

25 Villages

“Food+BCC”

30 kg rice

2 kg pulses

2ℓ cooking oil

BCC

500 Households

25 Villages

“Control”

497 Households

25 Villages

“Cash+Food”

750 Taka

15 kg rice

1 kg pulses

1ℓ cooking oil

499 Households

25 Villages

“Food only”

30 kg rice

2 kg pulses

2ℓ cooking oil

500 Households

25 Villages

“Cash only”

1,500 Taka

484 Households

556 Children 6-60m

487 Households

563 Children 6-60m

482 Households

551 Children 6-60m

489 Households

566 Children 6-60m

490 Households

539 Children 6-60m

14 Migrated

0 Refusal

1 Not traced

1 Incomplete data

10 Migrated

0 Refusal

0 Not traced

1 Incomplete data

10 Migrated

1 Refusal

1 Not traced

3 Incomplete data

3 Migrated

1 Refusal

2 Not traced

4 Incomplete data

9 Migrated

0 Refusal

0 Not traced

1 Incomplete data

2494 Households enrolled in study

6 Refusals
