# Supplementary material for: Behavior change communication activities improve infant and young child nutrition knowledge and practice of neighboring non-participants in a cluster-randomized trial in rural Bangladesh
Source: PLoS One. 2017 Jun 21;12(6):e0179866. doi: 10.1371/journal.pone.0179866 (PMC5479588; doi:10.1371/journal.pone.0179866)
Supplement: S1 Appendix — (PDF) [file pone.0179866.s006.pdf]

ClinicalTrials.gov PRS DRAFT Receipt (Working Version)
Last Update: 03/24/2015 04:58

ClinicalTrials.gov ID: NCT02237144

Study Identification

Unique Protocol ID: AEARCTR-0000247

Brief Title: Transfer Modality Research Initiative - Bangladesh (TMRI)

Official Title: Transfer Modality Research Initiative - Bangladesh

Secondary IDs:

Study Status

Record Verification: September 2014

⚠ WARNING : A record for an "Active, not recruiting" or "Enrolling by invitation" study must be reviewed, updated and verified at least once per year.

Overall Status: Active, not recruiting

Study Start: April 2012

Primary Completion: January 2015 [Actual]

Study Completion: December 2016 [Anticipated]

Sponsor/Collaborators

Sponsor: International Food Policy Research Institute

Responsible Party: Sponsor

Collaborators: United Nations World Food Programme (WFP)
German Ministry for Economic Cooperation and Development
Swiss Agency for Development and Cooperation
United States Agency for International Development (USAID)
Department for International Development, United Kingdom
CGIAR

⚠ WARNING : "German Ministry for Economic Cooperation and Development" is not a recognized organization name.

⚠ WARNING : "Swiss Agency for Development and Cooperation" is not a recognized organization name.

⚠ WARNING : "CGIAR" is not a recognized organization name.

Oversight

FDA Regulated?: No

IND/IDE Protocol?: No

Review Board: Approval Status: Approved

Approval Number: 03/18/2014
Board Name: Institutional Review Board (IRB IFPRI)
Board Affiliation: International Food Policy Research Institute
Phone: 2028624673
Email: ifpri-irb@cgiar.org

Data Monitoring?: No

Oversight Authorities: United States: Institutional Review Board

Study Description

Brief Summary: This is a randomized control trial in two zones of Bangladesh (north and south). Treatment is assigned at the village level where treatments are: cash transfers (north and south); cash transfers + nutrition behavior communication change (north only); food transfers (north and south); food transfers + nutrition behavior communication change (south only); food-cash split (north and south); and controls (north and south). Within treatment localities, women living in very poor households are targeted to receive benefits for two years.

Detailed Description: NOTE : Detailed Description: data not entered.

Conditions

Conditions: Improving Pre-school Anthropometric Status
Improving Household Food Security
Improving Maternal Nutrition Knowledge
NOTE : "Improving pre-school anthropometric status" is not a recognized condition
NOTE : "Improving household food security" is not a recognized condition
NOTE : "Improving maternal nutrition knowledge" is not a recognized condition

Keywords:

Study Design

Study Type: Interventional

Primary Purpose: Basic Science

Study Phase: N/A

Intervention Model: Factorial Assignment

Number of Arms: 5

Masking: Open Label

Allocation: Randomized

Endpoint Classification:

Enrollment: 5000 [Anticipated]

Arms and Interventions

Arms	Assigned Interventions
Experimental: Cash transfer	Cash transfer

Arms	Assigned Interventions
1500 taka ($18.75) per household distributed monthly	1500 taka ($18.75) per household distributed monthly
Experimental: Food transfer<br>30 kg rice, 2 kg lentils, and 2 kg micro-nutrient fortified cooking oil per household distributed monthly	Food transfer<br>30 kg rice, 2 kg lentils, and 2 kg micro-nutrient fortified cooking oil per household distributed monthly
Experimental: Food and cash transfer<br>15 kg of rice; 1 kg of lentils and 1 kg of micronutrient fortified cooking oil and 750 taka cash per household, distributed monthly	Food and cash transfer<br>15 kg of rice; 1 kg of lentils and 1 kg of micronutrient fortified cooking oil and 750 taka cash per household, distributed monthly
Experimental: Cash transfer + BCC<br>1500 taka ($18.75) per household distributed monthly Weekly, one hour meetings on maternal and child nutrition, sanitation and health knowledge, attitudes and practice Occasional home visits	Cash transfer<br>1500 taka ($18.75) per household distributed monthly<br>Behavioral: Behavior Communication Change (BCC)<br>Weekly, one hour meetings on maternal and child nutrition, sanitation and health knowledge, attitudes and practice Occasional home visits
Experimental: Food transfer + BCC<br>30 kg rice, 2 kg lentils, and 2 kg micro-nutrient fortified cooking oil per household distributed monthly Weekly, one hour meetings on maternal and child nutrition, sanitation and health knowledge, attitudes and practice Occasional home visits	Food transfer<br>30 kg rice, 2 kg lentils, and 2 kg micro-nutrient fortified cooking oil per household distributed monthly<br>Behavioral: Behavior Communication Change (BCC)<br>Weekly, one hour meetings on maternal and child nutrition, sanitation and health knowledge, attitudes and practice Occasional home visits

NOTE : Intervention 'Behavior Communication Change (BCC)' has not been included in any Arm/Group Descriptions.

Outcome Measures

Primary Outcome Measure:

1. Child anthropometry
   [Time Frame: Baseline. Up to 12 months. Up to 24 months.] [Safety Issue: No]
   Height for age z scores Weight for height z scores Weight for age z scores Stunting Wasting Outcomes specified in terms of levels one year after intervention started (2013), two years after intervention started and as changes: 2012-2013, 2013-2014 and 2012-2014

2. Child food intake
   [Time Frame: Baseline. Up to 12 months. Up to 24 months.] [Safety Issue: No]
   Number of unique foods consumed in previous 24 hours Number of food groups consumed in previous 24 hours Consumption of specified food groups - animal source foods, dairy, Vitamin A rich foods etc Caloric consumption in previous 24 hours Outcomes specified in terms of levels one year after intervention started (2013), two years after intervention started and as changes: 2012-2013, 2013-2014 and 2012-2014

3. Child health
   [Time Frame: Baseline. Up to 12 months. Up to 24 months.] [Safety Issue: No]
   Did mother report child was ill in previous 2 weeks Outcomes specified in terms of levels one year after intervention started (2013), two years after intervention started and as changes: 2012-2013, 2013-2014 and 2012-2014

4. Distribution of food intake within the household
   [Time Frame: Baseline. Up to 12 months. Up to 24 months.] [Safety Issue: No]
   Consumption of foods by all household members in previous 24 hours as recalled by mothers as measured by yes/no questions on foods, food groups and calories Outcomes specified in terms of levels one year after intervention started (2013), two years after intervention started and as changes: 2012-2013, 2013-2014 and 2012-2014

5. Household food security
   [Time Frame: Baseline. Up to 12 months. Up to 24 months.] [Safety Issue: No]
   Food groups consumed by household in previous seven days Number of unique foods consumed in previous seven days Number of food groups consumed in previous seven days Number of food groups consumed in previous seven days weighted by nutritional value and frequency of consumption Value of food consumption in previous seven days Household caloric availability in previous seven days Outcomes specified in terms of levels one year after intervention started (2013), two years after intervention started and as changes: 2012-2013, 2013-2014 and 2012-2014

6. Maternal nutrition knowledge
   [Time Frame: Baseline. Up to 12 months. Up to 24 months.] [Safety Issue: No]
   Maternal knowledge of infant and young child feeding practices (IYCF) Knowledge, attitudes, practice of IYCF Scored as yes/no and as a total score on all questions Levels assessed in 2013 and 2014 and as changes between 2012-2013, 2013-2014 and 2012-2014

Secondary Outcome Measure:

7. Household consumption
   [Time Frame: Baseline. Up to 12 months. Up to 24 months.] [Safety Issue: No]
   Value of food consumption in previous seven days Value of monthly non-food consumption Value of monthly non-food consumption by specified sub-groups (clothing, medical expenses, education expenses, transport, consumables, semi-durables) Value of monthly household consumption (food and non-food) Outcomes specified in terms of levels one year after intervention started (2013), two years after intervention started and as changes: 2012-2013, 2013-2014 and 2012-2014

8. Household income
   [Time Frame: Baseline. Up to 12 months. Up to 24 months.] [Safety Issue: No]
   Value of annual income derived from agriculture at endline (2014) Value of annual income derived from non-agricultural sources at endline (2014) Value of annual total income derived from all sources at endline (2014) Change in annual income (agriculture, non-agricultural, total) between 2012-2013, 2013-2014 and 2012-2014 Outcomes specified in terms of levels one year after intervention started (2013), two years after intervention started and as changes: 2012-2013, 2013-2014 and 2012-2014

9. Asset accumulation
   [Time Frame: Baseline. Up to 12 months. Up to 24 months.] [Safety Issue: No]
   Index (z score) of consumer durables Index (z score) of productive assets Index of quality of housing stock Productive land ownership (ha) Outcomes specified in terms of levels one year after intervention started (2013), two years after intervention started and as changes: 2012-2013, 2013-2014 and 2012-2014

10. Women's empowerment
    [Time Frame: Up to 12 months. Up to 24 months.] [Safety Issue: No]
    Index of women's decision making autonomy within the household Index of women's mobility outside the household Outcome measured as level in 2013, level in 2014 and change between 2013-2014

11. Information spillovers to non-beneficiaries
    [Time Frame: Up to 24 months.] [Safety Issue: No]
    Knowledge of infant and young child feeding practices (IYCF) of mothers Knowledge, attitudes, practice of IYCF Scored as yes/no and as a total score on all questions Non-beneficiaries are mothers living near intervention households (within approximately a 10 minute walk) in the following treatment arms: North: cash, cash + BCC, control. South: food, food + BCC, control. Levels assessed in 2014

NOTE : Normally only one Primary Outcome Measure is specified.

Eligibility

Minimum Age: 15 Years

Maximum Age: 65 Years

Gender: Female

Accepts Healthy Volunteers?: Yes

Criteria: Inclusion Criteria:

- Child age < 60 months

Exclusion Criteria:

- None

Contacts/Locations

Study Officials: Akhter Ahmed, PhD
Study Principal Investigator
International Food Policy Research Institute

John Hoddinott, DPhil
Study Principal Investigator
International Food Policy Research Institute

Shalini Roy, PhD
Study Principal Investigator
International Food Policy Research Institute

Locations: Bangladesh
International Food Policy Research Institute
Dhaka, Bangladesh

References

Citations:

Links:

U.S. National Library of Medicine | U.S. National Institutes of Health | U.S. Department of Health & Human Services
